# Wheat Leaf Antioxidative Status—Variety-Specific Mechanisms of Zinc Tolerance during Biofortification

**DOI:** 10.3390/plants10102223

**Published:** 2021-10-19

**Authors:** Ivna Štolfa Čamagajevac, Rosemary Vuković, Kristina Vuković, Ana Vuković, Vladimir Ivezić, Tanja Žuna Pfeiffer, Ljiljana Krstin, Zdenko Lončarić

**Affiliations:** 1 Department of Biology, Josip Juraj Strossmayer University of Osijek, Ulica Cara Hadrijana 8/A, HR-31000 Osijek, Croatia; istolfa@biologija.unios.hr (I.Š.Č.); avukovic@biologija.unios.hr (A.V.); tzuna@biologija.unios.hr (T.Ž.P.); lkrstin@biologija.unios.hr (L.K.); 2 Division for Molecular Medicine, Ruđer Bošković Institute, Bijenička cesta 54, HR-10000 Zagreb, Croatia; Kristina.Vukovic@irb.hr; 3 Department for Agroecology, Faculty of Agrobiotechnical Sciences Osijek, Josip Juraj Strossmayer University of Osijek, Ulica kralja Petra Svačića 1d, HR-31000 Osijek, Croatia; vladimir.ivezic@fazos.hr (V.I.); zloncaric@fazos.hr (Z.L.)

**Keywords:** zinc, lipid peroxidation, antioxidative status, wheat, biofortification

## Abstract

In this study, we evaluated the leaf antioxidative responses of three wheat varieties (Srpanjka, Divana, and Simonida) treated with two different forms of zinc (Zn), Zn-sulfate and Zn-EDTA, in concentrations commonly used in agronomic biofortification. Zn concentration was significantly higher in the flag leaves of all three wheat varieties treated with Zn-EDTA compared to control and leaves treated with Zn-sulfate. Both forms of Zn increased malondialdehyde level and total phenolics content in varieties Srpanjka and Divana. Total glutathione content was not affected after the Zn treatment. Zn-sulfate increased the activities of glutathione reductase (GR) and guaiacol peroxidase (GPOD) in both Srpanjka and Divana, while glutathione S-transferase (GST) was only induced in var. Srpanjka. Chelate form of Zn increased the activities of GST and GPOD in both Simonida and Divana. Catalase activity was shown to be less sensitive to Zn treatment and was only induced in var. Srpanjka treated with Zn-EDTA where GPOD activity was not induced. Concentrations of Zn used for agronomic biofortification can induce oxidative stress in wheat leaves. The antioxidative status of wheat leaves could be a good indicator of Zn tolerance, whereas wheat genotype and chemical form of Zn are the most critical factors influencing Zn toxicity.

## 1. Introduction

Zinc (Zn) is an essential metal for plants, and adequate availability of this micronutrient is vital at all stages of plant development. This micronutrient acts as an enzyme cofactor and thus plays an important role in regulating metabolic processes like the synthesis of nucleic acids and proteins, pollen formation, carbohydrate metabolism, and auxin synthesis [1,2]. However, excess of Zn may lead to phytotoxic effects that include disturbances in the uptake and translocation of nutrients, leaf chlorosis, reduced growth, and impairment of photosynthesis [3,4,5,6,7,8]. Moreover, excess of Zn, not being able to generate reactive oxygen species (ROS) directly through Haber–Weiss reactions, can indirectly induce oxidative stress in plants by different mechanisms such as activation of calcium-dependent systems, reduction of the glutathione (GSH) pool, and interruption of iron-mediated processes [9,10]. Zinc-induced ROS generation prompted oxidative injury in several plant organisms, causing different cellular effects such as lipid peroxidation, denaturation of proteins, and DNA mutations [3,4,11]. In response to heavy metal (HM) treatment, plant antioxidative enzymes can show induction of activity correlated with increased concentration of HMs, biphasic response, or inhibition of activity at high concentrations of HMs where plant species, genotype, and growth-stage are the most important factors influencing the HM toxicity [12]. Differential tolerance mechanisms of plant genotypes to Zn toxicity are a promising tool to complement our understanding of Zn tolerance in plants [13].

Zn fertilizers are widely used in agronomic biofortification to elevate Zn concentrations in grains of wheat and different cereals [14,15]. Additionally, Zn fertilizers can alleviate drought stress effects in wheat by reducing lipid peroxidation and increasing the content of photosynthetic pigments and active oxygen scavengers [16]. Namely, three different methods are used in traditional agronomic biofortification with Zn, including soil treatment, seed priming, and foliar application [17]. From all mentioned methods, foliar application of Zn fertilizers is the most beneficial due to low application rates, relatively high phloem mobility of Zn in cereals, and avoiding its losses through soil fixation [18]. Although agronomic biofortification proved to be a successful method to increase the concentration of Zn in wheat grain [19,20,21], the effects of different forms of Zn used in the biofortification on the whole plant level, especially on the antioxidative status in leaves, are not clearly elucidated. Several studies have shown that Zn-contaminated soils cause stress in plants [22,23], while Zn treatment induces oxidative stress and antioxidative response in wheat seedlings grown in Hoagland’s nutrient solution [24]. Therefore, more information is necessary concerning the effects of foliar-applied Zn on the antioxidative status of plants in field conditions. Antioxidative enzymes are susceptible to HM stress and give the fastest response, so they are very conducive biomarkers for assessing the toxic effects of foliar-applied Zn.

This work aimed to investigate the effects of two different forms of foliar-applied Zn on the leaf antioxidative status of three wheat varieties, var. Divana, a Croatian standard of high quality, var. Srpanjka, a standard for high yield and the most common variety in Croatia, and var. Simonida, a standard for high yield and the most common variety in agricultural production in Serbia. We hypothesized that the antioxidative response would be variety specific, and would be dependent on the applied Zn-form. The results given in this paper provide a better understanding of the possible toxicity of Zn used in agronomic biofortification and antioxidative leaf status as a potential biomarker of its toxicity. Additionally, this study emphasizes the importance of genetic factors of different wheat varieties that determines the variety-specific antioxidative response to Zn treatment.

## 2. Results

### 2.1. Zn Concentrations in Flag Leaves

Foliar application of Zn-sulfate did not cause changes in the Zn content in the flag leaves, while the treatment with Zn-EDTA significantly increased the concentrations of Zn in the flag leaves of all three wheat varieties compared to the control plants (Table 1). Variety Divana had the highest increase of Zn in the flag leaves, 14× higher than in control, while in var. Srpanjka and Simonida, the increase in Zn content was 11.6× and 5.3×, respectively.

### 2.2. Effects of Zn on the Products of Lipid Peroxidation

**Figure 1:**
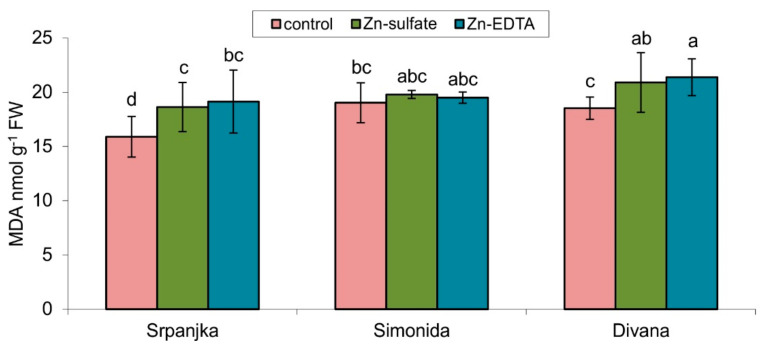
Content of malondialdehyde (MDA) in leaves of three different wheat varieties (Srpanjka, Simonida, Divana) treated with Zn-sulfate and Zn-EDTA. Results are presented as means ± standard deviation. Significant differences (*p* > 0.05) according to the LSD test were designated with a different letter (a, b, c, d).

The lipid peroxidation products, such as MDA, are usually measured as an indicator of oxidative stress. A significant increase in lipid peroxidation levels was showed in flag leaves of varieties Srpanjka and Divana treated with both forms of Zn (Figure 1). A slightly higher content of MDA was determined in leaves treated with Zn-EDTA in both varieties. The use of both forms of Zn, Zn-EDTA and Zn-sulfate, did not result in a significant change in the level of lipid peroxidation compared to control in the flag leaves of Simonida (Figure 1).

### 2.3. Effects of Zn on Total Glutathione Content and Related Enzymes

**Figure 2:**
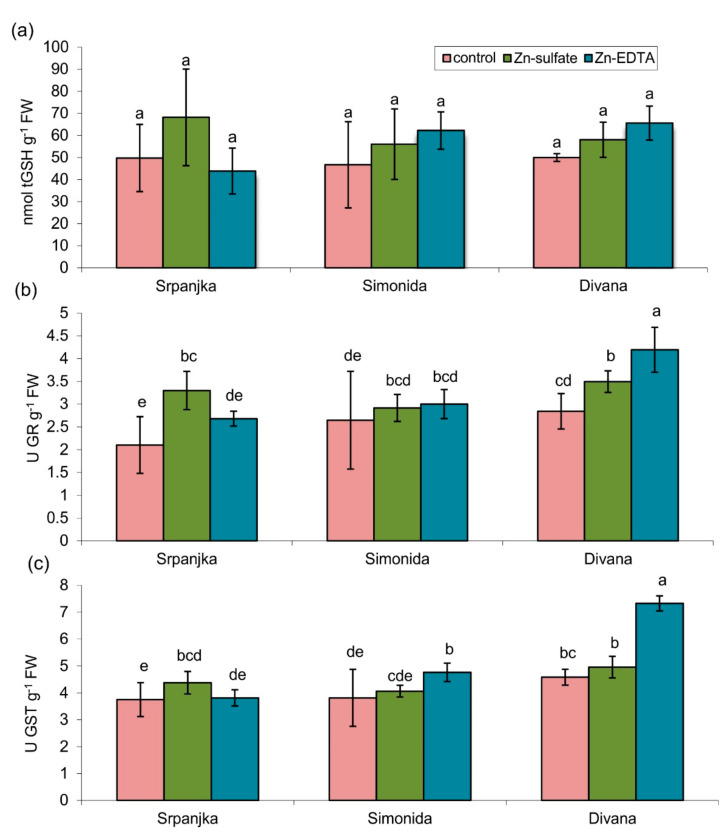
(**a**) Total glutathione (tGSH) content, (**b**) activity of glutathione reductase (GR), and (**c**) activity of glutathione S-transferase (GST) in leaves of three different wheat varieties (Srpanjka, Simonida, Divana) treated with Zn-sulfate and Zn-EDTA. Results are presented as means ± standard deviation. Significant differences (*p* > 0.05) according to the LSD test are designated with different letters (a, b, c, d, e).

In leaves of all three varieties tested, treatment with Zn in both forms had no impact on the total glutathione (tGSH) content (Figure 2a). The activities of glutathione reductase (GR) and glutathione S-transferase (GST) showed a similar trend (Figure 2b,c). The treatment with Zn-sulfate caused an increase in GR activity for 57% in leaves of var. Srpanjka and 25% in var. Divana compared to control (Figure 2b). In var. Divana, Zn-EDTA caused an increase in GR activity for about 50% compared to the control (Figure 2b). The activities of GST were significantly higher in vars. Simonida and Divana treated with Zn-EDTA while in var. Srpanjka, activity increased in leaves treated with Zn-sulfate compared to control (Figure 2c).

### 2.4. Effects of Zn on Antioxidant Enzyme Activities

**Figure 3:**
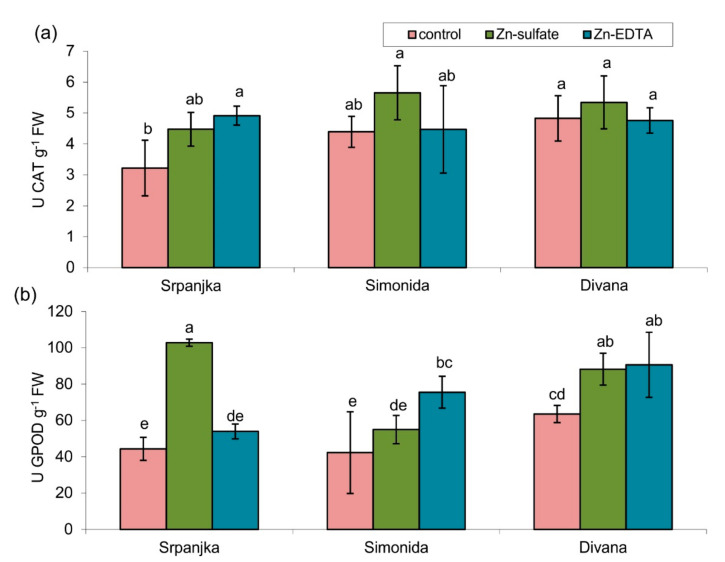
Activities of (**a**) catalase (CAT) and (**b**) guaiacol peroxidase (GPOD) in leaves of three different wheat varieties (Srpanjka, Simonida, Divana) treated with Zn-sulfate and Zn-EDTA. Results are presented as means ± standard deviation. Significant differences (*p* > 0.05) according to the LSD test are designated with different letters (a, b, c, d, e).

The antioxidant enzymes (guaiacol peroxidase (GPOD) and catalase (CAT)) activities were affected due to Zn treatment, although the effects depended on the wheat variety and the Zn form (Figure 3). The activities of CAT were not affected, except in leaves of var. Srpanjka treated with Zn-EDTA, where CAT activity was significantly higher than the control (Figure 3a). On the contrary, in the leaves of the same variety, Zn-sulfate caused a 132% increase in GPOD activity, while Zn-EDTA treatment had no effect (Figure 3b). In leaves of the other two varieties (Simonida and Divana), Zn-EDTA treatment caused an increase in GPOD activity compared to control, while Zn-sulfate had a significant effect only on the leaves of var. Divana (Figure 3b).

### 2.5. Effects of Zn on Soluble Phenolic Content

**Figure 4:**
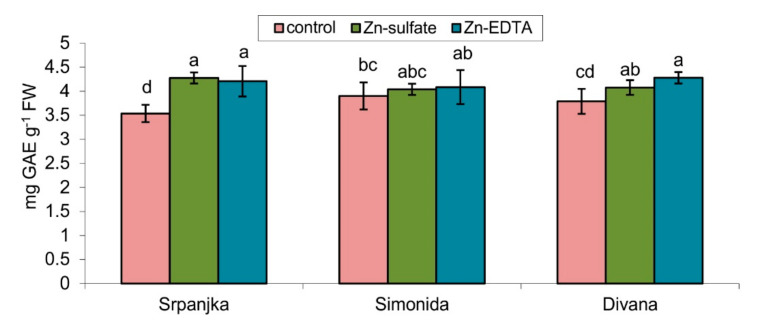
Total phenol content expressed in gallic acid equivalent (GAE) in leaves of three different wheat cultivars (Srpanjka, Simonida, Divana) treated with Zn-sulfate and Zn-EDTA. Results are presented as means ± standard deviation. Significant differences (*p* > 0.05) according to the LSD test are designated with different letters (a, b, c, d).

Treatments with both forms of Zn caused an increase in the total soluble phenolics content but only in leaves of var. Srpanjka and Divana. In the leaves of var. Simonida, Zn treatment did not cause significant changes in the content of total phenolics (Figure 4).

## 3. Discussion

Agronomic biofortification is a promising strategy for increasing Zn concentration in grains of different cereals [17]. Although foliar treatment with both forms of Zn caused an increase in the Zn content in wheat grains compared to control, Zn-sulfate was more effective than Zn-EDTA (unpublished results). Conversely, Zn concentration was significantly higher in the flag leaves of all three wheat varieties treated with Zn-EDTA compared to leaves treated with Zn-sulfate (Table 1). Similar results gained El-Nasharty et al. [25], who found that foliar-applied Zn-EDTA, compared to Zn-sulfate, generally has a greater impact on the Zn concentration in flag leaves and Zn use efficiency in shoots of different wheat varieties grown in calcareous soil. White and Broadley [26] established that the lower solubility of foliar-applied chelated Zn forms results in a higher degree of Zn retention on the leaf surface and/or in the apoplast, at which point the accumulated salts of Zn can interfere with many cellular processes. According to Nowack et al. [27], the increase in the Zn concentration in the stem after Zn-EDTA treatment does not necessarily mean a proportional increase in the concentration of Zn in wheat grain since the presence of EDTA blocks the transport of Zn from the stem to the grain.

Phytotoxic concentrations of Zn stimulate lipoxygenase activity leading to the induction of lipid peroxidation, one of the most reliable indicators of oxidative stress [28]. In this study, treatment with both forms of Zn caused a significant increase of lipid peroxidation level in the flag leaves of vars. Srpanjka and Divana compared to control plants, while Zn treatment did not cause the change in the MDA concentrations in flag leaves of var. Simonida (Figure 1). Panda et al. [29] established a positive correlation between Zn concentration and MDA amount in wheat leaves. Similar results have been reported in the leaves of Hyacinth bean (*Lablab purpureus*), and okra (*Hibiscus esculentus* cv. Hassawi) treated with Zn [30,31]. Although the leaves of the var. Simonida had a significantly higher concentration of Zn, no signs of oxidative stress in the leaves of this variety may result from more effective mechanisms responsible for the adoption and retention of Zn and an efficient antioxidative system. Increased capacity to maintain low levels of ROS due to increased activation of the antioxidant system is one of the primary mechanisms underlying the ability of sustainable growth and high productivity under environmental stress, and any genotype that has the capacity to do that would possess intrinsic tolerance to Zn [32,33]. Although treatment with Zn-EDTA resulted in a much higher concentration of Zn in flag leaves in all three varieties, this did not translate into a higher degree of oxidative stress or higher levels of antioxidative enzymes. An explanation for this effect can be found in the mitigation of heavy metal toxic effects by adding EDTA. This effect was previously observed in *Corchorus capsilaris* L. where the addition of EDTA diminished the copper toxic effects and, at the same time, improved copper accumulation Zn [34]. Diaz et al. [35] found similar toxicity mitigation results in pepper leaves treated with copper.

The results of studies conducted in different transgenic and wild-type plants, Zn accumulators, hyperaccumulators, and Zn-resistant plant genotypes, showed that higher levels of GSH and increased activity of enzymes involved in the metabolism of GSH result in intrinsic resistance to excess HMs [36,37,38]. GSH is a critical component of the antioxidant system and is involved in a wide range of physiological processes in the cell [39,40]. In addition to the antioxidant role, the nucleophilic nature of the thiol groups is particularly important in the detoxification of the redox-inert electrophiles such as Zn due to the direct conjugation of GSH with metal ions. Additionally, GSH is a direct substrate for phytochelatin synthesis that is important for maintaining a low concentration of free metal ions, especially in the case of an excess, thus protecting against potential oxidative damage to cellular structures [41,42].

In this research, Zn treatment did not cause significant changes in the GSH content compared to the control leaves (although GSH showed a tendency to increase in the leaves treated with Zn) (Figure 2a). On the other hand, several studies have shown that the relative proportion of GSH (reduced form) and oxidized GSH (GSSG), with simultaneous changes in the activity of GR is a reliable indicator of oxidative stress during exposure to the toxic effects of Zn [31,43,44]. Therefore, the ratio of GSH/GSSG would be a much better indicator of physiological status in leaves. Additionally, the duration of Zn exposure can be a relevant factor that can influence the GSH content. Panda et al. [29] showed that exposure to elevated concentrations of Zn could significantly change the metabolism of GSH in wheat leaves. According to their results, the acute treatment with Zn caused a slight increase of total GSH, while prolonged exposure to Zn resulted in a decrease of GSH content and the activity of antioxidant enzymes (CAT, superoxide dismutase, and GPOD), probably due to progression of oxidative stress [29].

In our study, foliar application of Zn-sulfate increased GR activity in the flag leaves of var. Srpanjka, while in var. Divana, both sources of Zn resulted in a significant increase in GR activity with a more pronounced effect of EDTA form (Figure 2b). On the contrary, Zn treatment had no significant effect on the GR activity in the flag leaves of var. Simonida (Figure 2b). Previous studies also point to the variety-specific differences in the activity of the GR in the leaves of different genotypes of pigeon pea (*Cajanus cajan*), rice (*Oryza sativa*), golden bean (*Vigna radiata*), and corn (*Zea mays*) treated with different HMs [45,46,47,48]. Furthermore, in wheat leaves treated with cadmium, two different isoforms of GR were determined [49]. The authors believe that the different times of activation and induction of various isoforms of the enzyme can result in modification of the total GR activity and a higher degree of GSH recycling over a prolonged period of stress. However, confirmation of this hypothesis requires further research [49]. The increased activity of the GR in the leaves, under conditions of high concentrations of Zn, is probably the result of the activities of the ascorbate-glutathione cycle [30], as confirmed by the analysis of the activities of GR and GSSG content in leaves of poplar exposed to elevated concentrations of Zn [44].

In our experiment, there was no correlation between the concentration of tGSH and GR activity, which was also found in hydroponic-grown seedlings of beans (*P. vulgaris*) exposed to elevated concentrations of Zn [43].

In addition to the ascorbate-glutathione cycle, high status of GSH is essential for the detoxification effect of GST. It was found that exposure to cadmium causes an increase GST activity in leaves of golden beans (*Vigna radiata*) [50], while Halusková et al. [51] showed in their study that increased activity of GST in barley (*Hordeum vulgare*) can be induced by a variety of metals, including Zn. As a stable and efficient response, GST activity is induced under conditions of increased oxidative stress when the basal antioxidant enzymes are exhaust [50].

Our results showed that increased activity of GST in the leaves might be the result of treatment with Zn (Figure 2c). In the leaves of var. Divana, Zn-EDTA caused an increase in the activity of GST for 37.5% compared to control (Figure 2c) what, with the increased level of lipid peroxidation (Figure 1), indicates that the increased activities of GST and GR were not sufficient to protect cells from oxidative damage caused by Zn. Increased GST activity in leaves of var. Simonida treated with Zn-EDTA and the absence of accumulation of MDA showed that in this variety adoption of Zn in the leaves did not reach the level of phytotoxicity. Zn ions are assumed to be transported to the vacuole through the GST pathway, avoiding their phytotoxic effect. In the leaves of var. Srpanjka treated with Zn-EDTA, there were no statistically significant changes in GST activity (Figure 2c), and it is possible that in this variety, GST was not primarily involved in the stress response induced by Zn-EDTA.

GPOD and CAT are the most important antioxidative enzymes in plants that detoxify H_2_O_2_ [52]. Their activities were induced by Zn treatment, but their induction depended on a wheat variety and the form of Zn (Figure 3). Unlike GPOD, CAT activity showed lower sensitivity to Zn treatment. Namely, CAT activity was only induced in leaves of var. Srpanjka treated with Zn-EDTA, where GPOD activity was not induced. The absence of CAT activity induction may be explained by its high capacity and low affinity for H_2_O_2_; thus, it is activated only when high levels of ROS are produced [7]. In addition to antioxidative enzymes, phenolics also have an important role in plant response to HM stress as non-enzymatic antioxidants or as a substrate for GPOD [53,54,55]. In our experiment, an increase in total phenolics content was also observed in leaves of vars. Srpanjka and Divana, with no changes in leaves of var. Simonida (Figure 4). An increase in total phenolics content is correlated with increased activities of GPOD in vars. Srpanjka and Divana, which can be connected with their role as a substrate for GPOD in detoxification of H_2_O_2_. An increase of total phenolics content was observed in different plant species exposed to HMs [35,56]. Ma et al. [16] found that in wheat leaves, Zn treatment elevates phenylalanine ammonia-lyase and chalcone synthase expression level, two crucial enzymes in the phenol biosynthetic pathway. Additionally, in wheat, nickel treatment caused an increase of soluble phenolics content [57], while the excess of aluminum caused lignin accumulation [58]. Such an increase in soluble phenolics, intermediates in lignin biosynthesis, could also reflect the higher cell wall resistance by making a physical barrier that prevents the entry of HMs [59,60]. In the case of var. Simonida, no significant increase in the content of soluble phenolics could be due to its embedment into cell walls of this variety. Moreover, var. Simonida had the smallest increase in the content of leaf Zn, suggesting a better cell wall resistance. Plants with higher content of phenolic compounds could have a greater potential to eliminate ROS and higher abilities to translocate and chelate heavy metals [60].

This research provides a better understanding of the wheat antioxidative defense mechanisms in response to Zn, and could contribute to the Zn-biofortification strategies and the sustainability of wheat production. In conclusion, we showed that concentrations of Zn used in agronomic biofortification can induce oxidative stress in wheat leaves. Leaf antioxidative response depends on the genotype and the chemical form of Zn used in the experiment. This research suggests that the antioxidative status of wheat leaves could be a reliable indicator of Zn tolerance, with var. Simonida being the most tolerant genotype. To understand the regulation of the processes of absorption, remobilization, compartmentalization, and transport of Zn to the grains, gene expression analysis of Zn-transporters should be performed.

## 4. Materials and Methods

### 4.1. Soils and Treatments

Three winter wheat (*Triticum aestivum* L.) varieties (vars. Divana, Simonida, and Srpanjka) were grown under field conditions in seasons 2012/13 on Mollic Gleysol in the Danube region (locality Banovci, Croatia). Sowing depth was 4–5 cm with seeding rate 220 kg/ha (Divana) or 260 kg/ha (Srpanjka and Simonida) seeds for 550 spikes/ha (Divana) or 700 spikes/ha (Srpanjka, Simonida). The Mollic Gleysol was calcareous (4.95% CaCO_3_), moderately alkaline (pH_H2O_ = 8.13), with moderate SOM (3.00%) total Zn content (53.5 mg kg^−1^) extracted by aqua regia (ISO, 1995), and plant available Zn content (1.11 mg kg^−1^) extracted by EDTA [61].

All treatments were distributed in complete randomized blocks with three replications of basic experimental plots (size 20 m^2^). The Zn foliar application treatments were as follows:

1. Control without Zn application;
2. Zn-sulfate: application of 1.5 kg ha^−1^ Zn in the form of ZnSO_4_ × 7 H_2_O (6.6 kg ha^−1^);
3. Zn-EDTA: application of 1.5 kg ha^−1^ of Zn in the form of Zn-EDTA (10 kg ha^−1^).

All treatments were applied using 600 L ha^−1^ of solutions (0.25% Zn *w*/*v*) or water for control treatment, plus 0.1% (*v*/*v*) surfactant, early in the morning between heading (Feekes 10.3) and beginning of flowering (Feekes 10.51). The leaves for analyses were collected by sampling across the whole plot for the average sample.

### 4.2. Analysis of the Zn Content

The dried leaf samples were ground into a fine powder using a heavy metal-free ultra-centrifugal mill (Retsch ZM 200). All samples were digested with 10 mL of HNO_3_ and H_2_O_2_ mixture (5:1, *v*/*v*) in a microwave oven (CEM Mars 6) at 180 °C for 60 min. The concentrations of Zn were determined by ICP-OES (PerkinElmer Optima 2100 DV) using an internal pooled plasma control and the reference material (Rice flour, IRMM-804, Sample No. 0533, European Commission, Joint Research Centre, Institute for Reference Materials and Measurements, Geel, Belgium) prepared in the same way as were the plant samples. All samples were analyzed in duplicate, and the results were expressed in mg kg^−1^ of dry weight.

### 4.3. Determination of the Products of Lipid Peroxidation

The products of lipid peroxidation in wheat leaves treated with Zn were determined with the method of Verma and Dubey [46] based on the measurement of the concentration of reactive substances of thiobarbituric acid, mainly malondialdehyde (MDA). About 200 mg of leaf tissue macerated with liquid nitrogen was extracted with 1 mL of 0.1% (*w*/*v*) solution of trichloroacetic acid (TCA). After centrifugation of the homogenates, the resulting supernatant was mixed with the thiobarbituric acid (0.5%, *w*/*v*) prepared in a 20% (*w*/*v*) TCA solution. The result of this reaction was the formation of red coloration, whose intensity was determined spectrophotometrically by measuring the absorbance at 532 nm and 600 nm. The absorbance at 600 nm was subtracted from the absorbance at 532 nm due to the correction for non-specific reactions. The amount of MDA was calculated based on the extinction coefficient (ε = 155 mM^−1^ cm^−1^) and expressed in nmol per g fresh weight (nmol MDA g^−1^ FW).

### 4.4. Measurement of Total Glutathione Content

Content of tGSH in wheat leaves treated with Zn was determined using a kinetic method based on a continuous reduction of 5,5-dithiobis (2-nitrobenzoic acid) (DTNB) to 5-thio-2-nitrobenzoic acid (TNB) by catalytic amounts of GSH, where GR and NADPH recycle the GSSG [62]. The formation of TNB was continuously recorded at 412 nm and 25 °C. Disrupted tissue was homogenized (1:10, *w*/*v*) in 5% 5-sulfosalicylic acid solution and centrifuged at 10,000× *g* for 10 min at 4 °C. The resulting supernatant was transferred to the reaction mixture that contained 100 mM phosphate buffer with 1 mM EDTA (pH 7.0), 0.031 mg mL^−1^ DTNB, and 0.115 units mL^−1^ of GR in a final volume of 1.05 mL. The reaction was initiated by adding NADPH at a final concentration of 48 μM. The total amount of GSH was determined by a standard curve of GSH, and the results were expressed as nmol g^−1^ of FW.

### 4.5. Extraction and Assays of Enzymes

Frozen leaf powder (about 200 mg) macerated with liquid nitrogen with the addition polyvinylpyrrolidone (PVP) was extracted for 15 min on ice with the addition of 1 mL of cold 100 mM potassium phosphate buffer (pH 7.0) containing 1 mM EDTA. Obtained homogenates were centrifuged for 15 min at 20,000× *g* and +4 °C. The resulting supernatants were stored at −80 °C and used for the spectrophotometric determination of the activity of the enzymes CAT (EC 1.11.1.6), GPOD (EC 1.11.1.7), GR (EC 1.8.1.7), and GST (EC 2.5.1.13).

The total CAT activity was measured by following the decrease in absorption at 240 nm for 2 min as H_2_O_2_ was catabolized [63]. The enzymatic reaction was started by adding 20 µL of enzyme extract in 1 980 µL of 50 mM potassium phosphate buffer (pH 7.0) with 10 mM H_2_O_2_. CAT activity was expressed in enzyme units (U) representing the amount of enzyme that catalyzes the oxidation of 1 µmol H_2_O_2_ per min at 25 °C and pH 7.0 per g of fresh weight.

The total GPOD activity was determined spectrophotometrically by measuring the absorbance increase at 470 nm. The reaction mixture contained 5 mM guaiacol and 5 mM H_2_O_2_ in 0.2 M phosphate buffer (pH = 5.8) [64]. The enzymatic reaction was started by adding 20 µL of the enzyme extract to 980 µL of the reaction mixture.

The activity of GR was determined according to the method described by Halliwell and Foyer [65]. The reaction mixture consisted of 400 µL of 100 mM potassium phosphate buffer (pH 7.5) containing 1 mM EDTA, 500 µL of 2 mM GSSG solution, 50 µL of crude enzyme extract, and 50 µL of 2 mM NADPH solution. The decrease in absorbance due to the oxidation of NADPH was monitored at 340 nm every 10 s for 2 min. GR was expressed in enzyme units (U) representing the amount of enzyme that catalyzes the oxidation of 1 µmol NADPH per minute at 25 °C and pH 7.5 per g of fresh weight.

The GST activity was conducted spectrophotometrically by monitoring the formation of the reaction product of conjugation between GSH and 1-chloro-2,4-dinitrobenzene (CDNB) at 340 nm [66,67]. The reaction was started by adding 50 µL of enzyme extract to the reaction mixture containing 1 350 µL of potassium phosphate buffer (pH 6.5) with 1 mM EDTA, 50 µL of 75 mM solution of GSH, and 50 µL 30 mM CDNB solution. Due to the formation of glutathione-2,4-dinitrobenzene conjugates, an increase in absorbance was measured every 30 sec for 5 min at 340 nm. One unit (U) of GST activity is equal to the amount of enzyme required for the conjugation of 1 µmol CDNB with GSH per min at pH 6.5 and 25 °C per g of fresh weight.

### 4.6. Extraction and Determination of Total Soluble Phenols

The ethanol extracts of wheat flag leaves were obtained by extracting 100 mg of frozen leaf powder with 1 mL of 80% ethanol in a bath at 80 °C for 30 min. The total phenol content was determined spectrophotometrically by the method of Folin-Ciocalteu [68]. The reaction mixture that consisted of 20 µL of ethanol extract, 1580 µL of dH_2_O, 100 µL of Folin-Ciocalteu reagent, and 300 µL of sodium carbonate saturated solution was incubated in a water bath at 37 °C for 1 h. The absorbance of the prepared samples was determined at 765 nm, and total phenol content in ethanol extract was calculated from the calibration curve with the gallic acid used as a standard. Soluble phenolic content in fresh tissue was expressed as mg of GA equivalents (GAE) per g of fresh weight.

## Figures and Tables

**Table 1:** Concentrations of Zn in leaves (mg kg^−1^ of dry weight) of three different wheat varieties (Srpanjka, Simonida, Divana) treated with Zn-sulfate and Zn-EDTA. Results are presented as means ± standard error. Significant differences between the treatments, in each variety separately, were determined using the LSD test (*p* ≤ 0.05), where differences were designated with a different letter (a, b).

Treatment	Srpanjka	Simonida	Divana
Control	12.14 ± 0.35 ^b^	23.59 ± 10.12 ^b^	13.05 ± 0.81 ^b^
Zn-sulfate	48.25 ± 20.22 ^b^	23.35 ± 5.36 ^b^	24.29 ± 3.57 ^b^
Zn-EDTA	152.43 ± 19.07 ^a^	148.00 ± 34.09 ^a^	194.98 ± 53.25 ^a^

## Data Availability

The dataset used and/or analyzed during the current study are available from the corresponding authors on reasonable request.
